# Needle-Based Arthroscopic Transosseous Rotator Cuff Repair: A Short-Term Outcomes Analysis

**DOI:** 10.7759/cureus.13595

**Published:** 2021-02-27

**Authors:** Ehud Atoun, John G Horneff, Ofer Levy, Walter Stanwood, Nikhil Verma, Joseph A Abboud

**Affiliations:** 1 Orthopaedics, Barzilai Medical Center, Ashkelon, ISR; 2 Shoulder and Elbow Surgery, Rothman Institute, Thomas Jefferson University, Philadelphia, USA; 3 Shoulder and Elbow Surgery, Reading Shoulder Unit, Royal Berkshire Hospital, Reading, GBR; 4 Orthopaedics, Plymouth Bay Orthopaedics, Duxbury, USA; 5 Orthopaedics, Midwest Orthopaedics at Rush University, Chicago, USA

**Keywords:** rotator cuff tear, transosseous repair, outcome scores, arthroscopic shoulder surgery

## Abstract

Introduction

Given the limitations of anchor-based rotator cuff repair, surgeons have considered and investigated the use of an arthroscopic transosseous repair technique using only sutures to repair tendon tissue. Returning full circle to the gold standard of transosseous repair, but with the modern adaptation of arthroscopy, advocates of arthroscopic transosseous rotator cuff repair believe that many of the risks associated with suture anchors can be avoided. The purpose of this study was to examine the capabilities of a novel needle-based arthroscopic transosseous tunneling device (OmniCuff™ arthroscopic transosseous device, MinInvasive Ltd., Magal, Israel) and evaluate the short-term clinical outcomes and patient satisfaction of patients treated with this technique.

Materials and methods

This study was a prospective, single-arm, multi-center study performed on patients from January 2014 to March 2015. During the study period, thirty-two patients underwent arthroscopic transosseous rotator cuff repair using the OmniCuff™ arthroscopic transosseous device.

Results

The average age of patients was 58.2 years (range, 44 to 80 years). The sizes of the tears were as follows: seven small, 18 medium, four large, and three massive. The average number of tunnels used per repair was 1.9 with the following breakdown: six one-tunnel repairs, 22 two-tunnel repairs, and four three-tunnel repairs. The mean American Shoulder and Elbow Surgeon (ASES) score improved from 45.1 to 87.7, the mean Simple Shoulder Test (SST) score improved from 42.6 to 92. Overall patient satisfaction was high with an average Likert scale of 4.6.

Conclusion

Our study demonstrated significantly improved outcomes for patients undergoing arthroscopic transosseous rotator cuff repair using the needle based Omnicuff device. Patients were overall very satisfied with the outcome of their surgery and their ASES and SST scores demonstrated this appropriately.

## Introduction

Since its description by Codman in 1911 and long before the technology of arthroscopy was available, the gold standard treatment of rotator cuff (RC) tears was an open transosseous (TO) repair [1]. The advent of arthroscopic techniques enabled the development of arthroscopic rotator cuff repair (RCR), which offers the advantages of smaller skin incisions, minimal soft tissue dissection, access to additional shoulder pathologies in the glenohumeral joint, and preservation of the overlying deltoid muscle [2-4]. Since at the early stages of arthroscopic RCR, an arthroscopic transosseous (ATO) repair was not technically feasible, surgeons became adapt to performing the majority of all-arthroscopic repairs with the use of suture anchors. Nevertheless, regardless of which type of suture anchor configuration is preferred, there are inherent limitations to the use of anchors for RCR.

When placed, suture anchors occupy space within the limited rotator cuff footprint area. This area is critical for the development of an inflammatory response that initiates the cascade of tendon-to-bone healing. Furthermore, anchors decrease the blood flow to the footprint area as compared to bone tunnels, further inhibiting the tendon-to-bone healing process [5]. Third, if failure of the repair occurs, the space occupied by the anchors decreases the available footprint in an already depleted area. Fourth, if an anchor dislodges from its insertion site, the patient is left with a loose body that can become painful or even damage the surrounding structures within the shoulder joint. In addition, the use of absorbable anchors has been associated with complications, including foreign-body reactions, cyst formation, fluid collection, sterile drainage, osteolysis, and chondral damage [6]. Lastly, a recent study demonstrated that the cost of transosseous-equivalent (TOE) RCR is significantly higher than the cost of ATO [7]. All of these concerns are amplified as the number of anchors used in a repair increases.

Given these limitations of anchor-based RCR, surgeons have considered and investigated the use of an ATO repair technique using only sutures to repair tendon tissue [7-9]. Returning full circle to the gold standard of repair, initially reported by Codman, but with the modern adaptation of arthroscopy, advocates of ATO RCR believe that many of the aforementioned risks associated with suture anchors can be avoided entirely.

The purpose of this study was to examine the capabilities of a novel needle-based ATO tunneling device (OmniCuff™ arthroscopic transosseous device, MinInvasive Ltd., Magal, Israel), and evaluate the short-term clinical outcomes and patient satisfaction of patients treated with this technique.

## Materials and methods

This study was a prospective, single-arm, multi-center study performed on patients from January 2014 to March 2015. The respective Institutional Review Boards at each institution approved this study. During the study period, 32 patients underwent ATO RCR using the OmniCuff™ arthroscopic transosseous device (MinInvasive Ltd., Magal, Israel) as previously described [10].

Any males and non-pregnant females 18 years of age or older with a full-thickness rotator cuff tear that was deemed repairable by MRI were included in the study. Patients with irreparable tears, subscapularis tendon tear, or those with no evidence of an RC tear upon arthroscopic evaluation were excluded. Lastly, any patients with prior surgery, fracture, or dislocation of the operative shoulder were excluded. All patients were asked to fill out questionnaires prior to surgery and at the latest follow-up of at least one year. 

The primary outcome measures were the American Shoulder and Elbow Surgeon (ASES) self-reported score and Simple Shoulder Test (SST) score. In addition, at the most recent follow-up, patients were asked to rate the overall satisfaction with their surgery outcome on a Likert scale of 1 to 5 (1=very unsatisfied; 2=somewhat unsatisfied; 3=neutral; 4=somewhat satisfied; 5=very satisfied). Demographic and surgical information was also collected for analysis of contributing factors including age, sex, size classification of the tear (small [<1 cm], medium [1-3 cm], large [3-5 cm], massive [>5 cm]), the actual size of the tear (in centimeters), number of transosseous tunnels used, and number of sutures used per tunnel. Post-operative rehab progressed in the usual manner with sling immobilization for four-six weeks followed by the initiation of passive range of motion (PROM) exercises at four-eight weeks, active range of motion (AROM) exercises at 8-12 weeks and incorporation of strengthening from weeks 12-20. The patient evaluation was performed by an independent observer pre-operatively and post-operatively, 3, 6, 9, 12, 18, 24, and 36 months post-operatively.

## Results

Thirty-two patients were included in this study. There were 10 females (31.3%) and 22 males (68.8%). The average age of patients was 58.2 years (range, 44 to 80 years). The sizes of the tears were as follows: seven small, 18 medium, four large, and three massive. The average number of tunnels used per repair was 1.9 with the following breakdown: six one-tunnel repairs, 22 two-tunnel repairs, and four three-tunnel repairs. The number of sutures used per tunnel ranged from two to four, with the majority of tunnels using three sutures (43/62; 69.4%). Of the 32 patients included in the study, 28 (87.5%) were available for final follow-up at an average of 24.7 months from surgery (range, 15.7 to 29.4 months). Two of these patients went on to revision surgery for re-tear of the rotator cuff and therefore were not included in the analysis of post-operative scores. Of the two patients undergoing revision surgery, one patient did not demonstrate improvement following surgery, while another re-injured the shoulder when lifting a heavy grocery bag nine months following surgery. This left 26 patients available for post-operative analysis. Besides the two re-tears, there were no other complications encountered. 

Twenty of the 26 patients (76.9%) who were available for the latest follow-up also had pre-operative ASES and SST scores recorded. The mean pre-operative ASES score was 45.1. The mean pre-operative SST score was 42.6. 

The mean post-operative ASES score was 87.7 and the mean post-operative SST score was 92. When the post-operative scores were averaged for only the 20/26 patients with available pre-operative scores, the improvement between and pre- and post-operative scores was even more pronounced. For those patients with recorded pre-operative scores, the mean post--operative ASES score was 88.4 and the mean postoperative SST score was 92.5.

**Figure 1:**
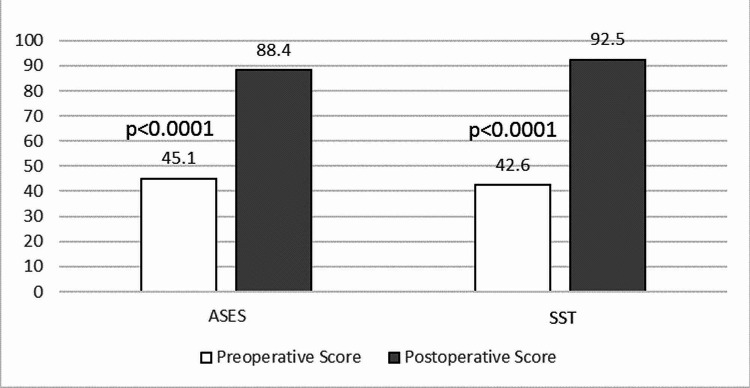
Outcome Mean pre-operative and post-operative shoulder scores comparison following MinInvasive OmniCuff™ rotator cuff repair ASES: American Shoulder and Elbow Surgeon score; SST: Simple Shoulder Test score

Both ASES and SST postoperative scores displayed significant improvement at the latest follow-up compared to pre-operative scores for the 20 patients with complete data. The mean ASES improved from 45.1 pre-operatively to 88.4 post-operatively (p<0.0001; SD: 18.5). The mean SST improved from 42.6 pre-operatively to 92.5 post-operatively (p<0.0001; SD: 24.3) (Figure 1).

Using the Likert scale of patient satisfaction, the overall patient satisfaction was high with an average Likert scale of 4.6. Of the 26 patients who were available for the latest follow-up, 19 patients (73%) declared a score of ‘5’, indicating that they were very satisfied with their surgical outcome. Only three patients (11.5%) declared a score of ‘3’, indicating a neutral level of satisfaction regarding their surgical outcome. Two of these patients had medium-sized tears and one had a massive-sized tear. All three of these patients were treated with a 2-tunnel repair. None of the patients declared a level of dissatisfaction (score of 1 or 2) with their surgery.

## Discussion

Arthroscopic rotator cuff repair is one of the most commonly performed orthopaedic procedures in the United States with more than 650,000 cases performed annually [11,12]. Colvin et al. reported that rotator cuff repairs account for 98 procedures per 100,000 people in the United States in 2006 [13]. The majority of these arthroscopic repairs utilize suture anchors that are inserted into bone with each anchor providing a single point of fixation for the tendon tissue. Although popular because of their relative ease and speed of use, suture anchors for rotator cuff repair have various shortcomings that affect multiple facets of patient care including surgical morbidity and economic impact. These concerns have fueled a trend in the literature supporting ATO RCR with the use of suture through bone tunnels. 

Recent studies have supported the use of transosseous RCR. These techniques have been found to offer several advantages on anchor repair including a broad footprint coverage with no foreign body at the footprint area, lower risk of RCR failure at the musculotendinous junction (type 2 failure) [14], lower risk of Type III Sugaya MRI readings (insufficient thickness after repair) [15], improved blood flow at the bone-tendon interphase [5] and decreased pain with similar functional outcomes [16].

Kuroda et al. performed a series of 384 patients with transosseous RCR and displayed very satisfactory results [17]. Patients improved from a mean preoperative University of California, Los Angeles (UCLA) Shoulder Rating Scale of 19.1 to a mean postoperative score of 32.7. Only 6% of patients experienced a re-tear, which is lower than many reports of anchor-based repairs [17-20]. In the study by Kuroda et al., the authors had excluded large RC tears and stated that this may have accounted for the relatively low re-tear rate. 

Although we used different shoulder scoring systems, both ASES and SST scores in our series showed statistically significant improvements (ASES, p<0.0001; SST, p<0.0001). In our series, only two patients (7.1%) suffered a re-tear, which is a similar rate to the study by Kuroda et al. Both of these failures occurred in medium-sized tears that would not have been excluded in the aforementioned study. One patient continued to have pain throughout post-operative physical therapy, which led to a post-operative MRI confirming a re-tear; the other patient had been progressing well after surgery but noted increased symptoms after an episode of lifting a heavy grocery bag with the operative arm about nine months after surgery. The fact that our study included large and massive tears that did not go on to failure demonstrates that the concern expressed by Kuroda et al. of failure in larger tears may not be as significant as they considered. 

A recent cost-analysis study by Black et al. compared anchorless TO RCR with TOE repair that requires the use of double-row anchors. The authors of that study found that the average cost of an anchored repair was $1014.10 compared to an average cost of $678.45 for an anchorless repair [7]. This is certainly a significant consideration for patients, manufacturers, payers, and hospitals alike. The authors found that the cost difference increased in correlation with tear size, with the fluctuation in price varying as much as $694.29 on average for anchor implants [7]. Conversely, the average difference in price for tunnel repair was only $56.30, with the patients in that study averaging seven sutures per repair. 

Lastly, complications of surgery are always a huge consideration when deciding on a technique. As previously stated, Kuroda et al. described a re-tear rate of 6% [17]. They also described only one complication with breakage of the bone bridge during surgery in one patient [17]. In another study, Black et al. examined 31 patients who underwent TO RCR. They had a re-tear rate of 9.7%, which they labeled as a “major complication” [8]. The authors also reported a minor complication rate of 6%, with both cases including suture cutout through the bone bridge similar to the Kuroda study [8]. We had no minor complications in our patient cohort. 

This study did have several limitations. Given that this was a case series, there was no comparative group of patients undergoing anchor-based repair. In addition, although we did achieve a follow-up rate of 87.5%, two patients were lost to follow-up, thereby decreasing the power of our study. Lastly, although all patients included in the study had a minimum follow-up of one-year, a longer follow-up would be beneficial.

In conclusion, the MinInvasive OmniCuff™ transosseous rotator cuff repair system demonstrated significantly improved outcomes for treated patients. Patients were overall very satisfied with the outcome of their surgery and their ASES and SST scores demonstrated this appropriately. Complication rates on both the major and minor scales were comparable to data in the literature. 

Anchor-based rotator cuff repair is fraught with limitations that only increase as more anchors are used to recreate a transosseous-equivalent repair. The use of a transosseous cuff repair system that uses bone tunnels rather than anchors to secure the tendon to bone is certainly advantageous when attempting to recreate the anatomical footprint of the rotator cuff. This is especially true in the case of large tendon tears and revision repairs. 

## Conclusions

Arthroscopic TO rotator cuff repair using the OmniCuff™ device leads to significant short-term improvement in patient satisfaction, ASES and SST scores in patients with various-sized rotator cuff tears. This technique could be a viable option in the treatment of rotator cuff tear, but further studies need to be performed to evaluate clinical and radiographic outcomes with this technique.
